# The Impact of Illness Perceptions and Coping Strategies on Use of Supportive Care for Cancer

**DOI:** 10.3390/cancers13102501

**Published:** 2021-05-20

**Authors:** Peta Stephenson, Eva Yuen, Gemma Skaczkowski, Evelien R. Spelten, Sheina Orbell, Carlene Wilson

**Affiliations:** 1 Department of Psychology and Counselling, School of Psychology and Public Health, La Trobe University, Bundoora, VIC 3083, Australia; petaks@hotmail.com (P.S.); e.yuen@deakin.edu.au (E.Y.); Gemma.Skaczkowski@unisa.edu.au (G.S.); 2 Psycho-Oncology Research Unit, Olivia Newton-John Cancer Wellness and Research Centre, Austin Health, Heidelberg, VIC 3084, Australia; 3 School of Nursing and Midwifery, Faculty of Health, Deakin University, Melbourne, VIC 3125, Australia; 4 Centre for Quality and Patient Safety Research–Monash Health Partnership, Institute for Health Transformation, Deakin University, Melbourne, VIC 3125, Australia; 5 Allied Health and Human Performance, University of South Australia, Adelaide, SA 5001, Australia; 6 Department of Rural Health, La Trobe Rural Health School, La Trobe University, Bundoora, VIC 3083, Australia; e.spelten@latrobe.edu.au; 7 Department of Psychology, University of Essex, Colchester CO4 3SQ, UK; sorbell@essex.ac.uk

**Keywords:** survivorship support programmes, supportive care, oncology care, common sense model of self-regulation, Leventhal, illness perceptions, coping

## Abstract

**Simple Summary:**

Cancer survivorship support programmes improve wellbeing, but most oncology patients and survivors do not use them. The current study examined whether people who saw themselves as having greater personal control over their illness used more problem-focused coping strategies, and if this resulted in higher use of survivorship support services. The study also examined the possibility that use of supportive care services was higher amongst those patients who used more emotion-focused coping strategies and who were experiencing greater emotional distress about their cancer. We found that people using more survivorship support services tended to have a greater belief in their ability to do something to control their cancer but were not emotionally distressed.

**Abstract:**

Despite evidence that survivorship support programmes enhance physical and psychosocial wellbeing, cancer patients and survivors often do not use these supportive care services. This study investigated the utility of the Common Sense Model of Self-Regulation for predicting supportive care use following cancer, and the mediating role of coping strategies. Cancer patients and survivors (*n* = 336 from Australia, *n* = 61 from the UK; 191 males, 206 females) aged 20–83 years (Mean (M) = 62.73, Standard Deviation (SD) = 13.28) completed an online questionnaire. Predictor variables were cognitive and emotional representations of cancer, as measured by the Illness Perception Questionnaire—Revised (IPQ-R), and problem- and emotion-focused coping strategies, as measured by the Brief-Coping Orientation to Problems Experienced inventory (Brief-COPE). The outcome variable was survivorship support programme use within the preceding month. Perceived personal control over cancer predicted supportive care use, but cancer-related emotional distress did not. Coping was an inconsistent mediator of the relationships. Problem-focused coping mediated the relationship between personal control and supportive care use; emotion-focused coping did not mediate between emotional responses to cancer and the uptake of survivorship support programmes. The Common Sense Model provides a useful framework for understanding survivorship support programme use. However, more clarity around the relationship between illness beliefs and coping is required.

## 1. Introduction

Recent studies reported that over one-third of people with cancer exhibit significant levels of psychological distress [1,2]. Social support has been identified as instrumental in helping cancer patients cope [3]. Engaging with survivorship support services, including cancer helplines, joining professionally led peer support groups, attending educational seminars or meeting with social workers, therapists, counsellors, chaplains and spiritual advisors has been shown to reduce patients’ emotional distress and improve wellbeing [4,5]. Survivorship supportive care is understood as a multidimensional, person-centred approach to delivering essential services for people with cancer to help them fulfil their physical, psychological, practical, informational, spiritual and social needs during cancer diagnosis, treatment and survivorship [4]. Patient evaluations of cancer survivorship support programmes, whether offered in a hospital or an outpatient setting, indicate multiple benefits including reduced stress levels, more energy, better sleep, and elevated hopefulness and empowerment [6]. However, many oncology patients do not take advantage of supportive care services [7].

This finding is likely repeated with other health conditions. A study examining the predictors of participation in a chronic disease self-management (CDSM) programme indicated that physical ill health compromises participation [8]. Male gender is also associated with poorer participation in CDSM, although the results suggested that different venues and modes of delivery may improve participation [9]. Better knowledge of the factors that predict engagement with survivorship support programmes is important, given the potentially deleterious effects of non-engagement for patient wellbeing and survivorship. The variables found to best predict supportive care use have the potential to be used as the basis for developing interventions designed to increase patient engagement with psychosocial support programmes [10].

Leventhal and colleagues’ Common Sense Model of Self-Regulation predicts self-care responses to chronic and life-threatening illness [11,12,13]. According to the model, people actively construct their own common-sense pattern of illness beliefs in their efforts to deal with the impacts of disease [11,14]. The model proposes that people use two related pathways to adapt to illness. The first involves a cognitive perception or representation of the illness and the associated coping response to manage the threat; the second pathway includes an emotional representation of the illness and a corresponding coping response to manage the emotional distress associated with the threat [10,15]. Further, feedback loops operate in which individuals assess the effectiveness of their coping attempts, and these appraisals then modify their cognitive and emotional illness representations which, in turn, influence subsequent coping procedures.

According to the Common Sense Model, illness perceptions are associated with coping strategies and, via these, with behavioural outcomes [16]. The proposition is that coping mediates the relationship between an individual’s illness representations and their self-care response [10,17]. Although the Common Sense Model does not specify particular types of coping, a number of theoretically derived coping dimensions have been used in the literature that broadly correspond to those proposed by Folkman and Lazarus [18]. Folkman and Lazarus distinguished between problem-focused coping (an active process of problem-solving designed to remove the threatening external event) and emotion-focused coping (aimed at managing the internal distress associated with the threat). Problem-focused coping represents the use of strategies to manage a stressful situation (e.g., seeking information, making plans), while emotional-focused coping describes strategies used to regulate emotions caused by an event or situation (e.g., information avoidance, social support seeking, etc.).

Grande et al. [19,20] investigated cancer patients’ attitudes to joining cancer support groups. They found that higher participation was associated with favourable beliefs about support groups and greater perceived personal control over cancer. Building on this research, the current study investigated illness perceptions and coping as predictors of oncology patients’ engagement with survivorship support programmes in general. According to Carver et al. [21], people seek supportive care for two main reasons: seeking social support to gain advice, assistance or information is akin to problem-focused coping; seeking social support for understanding or emotional or moral support is congruent with emotion-focused coping.

As indicated above, the relationship of illness perceptions to the seeking and use of supportive care is likely to operate through both cognitive and affective pathways. Specifically, those who deal best with illness by adopting strategies to enhance perceived personal control may cope with the challenges of cancer by seeking professional support. Simultaneously, those with strong emotional representations that present as anxiety or fear may seek support to deal with these negative responses. Based on the above, a number of predictions can be made about the relationship among personal control, emotional representations and participation in supportive care, and the mediating role of different strategies for coping. Specifically, consistent with past research, we predicted that those with higher levels of personal control over cancer would report reliance on more active, problem-focused coping strategies [22,23,24] and that this would be associated with greater use of survivorship support services than those with lower perceived personal control. Additionally, participants with strong emotional representations of cancer would use emotion-focused coping strategies and be more likely to utilise supportive care than those with lower cancer-related emotional distress. These two alternative pathways to survivorship supportive care reflect an effort, on one hand, to manage the external stressor itself and, on the other, to manage the associated inner turmoil [21].

The aim of the current study was to assess the extent to which personal control, emotional representations and coping strategies predict cancer patients’ engagement with survivorship support programmes. Based on previous research, it was hypothesised that the probability of supportive care use would be predicted by the following four independent variables: greater perceived personal control of illness, greater adoption of problem-focused coping strategies, higher cancer-specific emotional representations and greater adoption of emotion-focused coping strategies. Assuming that these hypotheses are supported, it was further predicted that use of problem-focused coping strategies would mediate the relationship between personal control and survivorship supportive care use, and that the use of emotion-focused coping strategies would mediate the relationship between emotional representations and supportive care use.

## 2. Materials and Methods

### 2.1. Participant Recruitment and Eligibility Criteria

Participants were recruited through the market research company Prolific (Oxford, UK) using a two-step process. In the first step, a screening survey was distributed to identify individuals who met the eligibility criteria. Individuals who were diagnosed with cancer in the preceding 5 years, aged 18 years or older, resided in Australia or the UK, and able to read and write English were eligible to participate. Those with early-stage melanomas or non-melanoma skin cancers were excluded, as these cancers may follow a different treatment pathway (e.g., day surgery) and patients may not typically have the same experience of cancer treatment as those with other cancer diagnoses. Individuals who met the eligibility criteria were then sent the full survey to their anonymous Prolific account to complete. Participants were reimbursed 13c following completion of the screening survey and AUD 10 following completion of the full survey. A description of participants is provided in Table 1. The study was approved by the Human Research Ethics Committee at La Trobe University, Melbourne, Australia (HEC18534).

### 2.2. Materials

The online questionnaire collected the following data from participants: Demographic information, including age, gender, place of residence, postcode, country of birth, preferred language, highest level of education, marital status, cancer diagnosis type, and month and year of cancer diagnosis.

Predictor variables (perceived personal control and emotional representations): Two subscales from the Illness Perception Questionnaire—Revised (IPQ-R) [25] were used to assess perceived personal control (6 items, e.g., “There is a lot which I can do to control my symptoms.”) and emotional representations (6 items, e.g., “I get depressed when I think about my cancer.”). The IPQ-R was developed to examine individual differences in people’s responses to chronic illness and has demonstrated adequate validity and reliability across chronic disease populations [26]. Given that the current study aimed to assess the relationship between patients’ thoughts and feelings about cancer and their uptake of survivorship supportive care services, only the personal control and emotional representations subscales of the IPQ-R were utilised. As the IPQ-R is a generic instrument designed for use with various health conditions, in the present study, the word “illness” was replaced with “cancer”. The level of agreement with each statement was rated on a 5-point Likert-type scale from 1 (strongly disagree) to 5 (strongly agree). Items for each subscale were summed to create an overall score. Higher scores on the personal control subscale represent greater belief about one’s controllability of the cancer. Higher scores on the emotional representations subscale reflect greater impacts of an individual’s illness on their emotional wellbeing. In the current study, Cronbach’s alphas of 0.85 and 0.93, respectively, were reported, indicating good internal consistency.

Predictor variables (coping): The Brief-Coping Orientation to Problems Experienced (Brief-COPE) [27] inventory was used to assess the coping strategies used (emotion-focused and problem-focused). The Brief-COPE is a well validated and frequently used measure of coping [28]. It assesses the frequency with which an individual uses different coping strategies on a 4-point Likert-type scale from 1 (“I haven’t been doing this at all.”) to 4 (“I’ve been doing this a lot.”). Two subscales from the Brief-COPE were included in the analysis: problem-focused coping strategies (6 items) and emotion-focused coping strategies (10 items). The problem-focused coping strategies subscale assessed an individual’s efforts to diminish the impact of their cancer and comprised items that assessed active coping, planning and use of instrumental support. The emotion-focused coping strategies subscale assessed an individual’s efforts to reduce the negative feelings associated with having cancer, and comprised items measuring acceptance, use of emotional support, humour, positive reframing and religion. Higher scores on each subscale reflect greater use of the specific coping strategy. In the current study, Cronbach’s alphas of 0.84 and 0.77, respectively, were reported.

Outcome variable: Survivorship support service use in the preceding month was measured using a single item “Have you attended any supportive care programmes in the past month?” The response option to the item was dichotomous (yes/no).

### 2.3. Data Analysis

SPSS v27 [29] was used for data analysis. Independent sample *t*-tests compared the average time since diagnosis in months and age in years reported by participants who did and did not use supportive care. Pearson’s chi-square tests of contingencies evaluated whether the participants’ gender, marital status and level of education were related to their use or non-use of survivorship support programmes. Additional independent sample *t*-tests examined whether participants’ personal control, emotional representations, problem-focused coping and emotion-focused coping scores differed between supportive care users and non-users. Binary logistic regression analyses assessed whether personal control and problem-focused coping predicted uptake of survivorship supportive care, and if emotional representations and emotion-focused coping predicted the probability of using supportive care. These analyses controlled for demographic variables found to be significant in previous analyses: age, gender, time since diagnosis, marital status and place of residence (Australia, UK). Bivariate Pearson’s product–moment correlation coefficients assessed the size and direction of the linear relationships between the two illness perception variables (personal control and emotional representations) and the two coping variables (problem-focused and emotion-focused). A mediation model was run using PROCESS macro v3.5 [30] to predict supportive care use from personal control via problem-focused coping.

## 3. Results

### 3.1. Participant Overview

Of the initial sample of eligible participants (*n* = 459), 62 cases were excluded because of extensive missing data. Of the remaining *N* = 397 participants, *n* = 336 were from Australia and *n* = 61 from the UK. Participants were aged 20–83 years (M = 62.73, SD = 13.28).

### 3.2. Group Differences in Demographic Variables and Self-Reported Personal Control, Emotional Representations, Problem-Focused Coping and Emotion-Focused Coping

Pearson’s chi-square compared users of survivorship support programmes (*N* = 97) with non-users (*N* = 300) on gender, marital status, level of education and place of residence (Australia, UK). Significant differences were found for gender, (χ^2^ (1, *N* = 397) = 11.87, *p* = 0.001), with a greater proportion of women reporting supportive care use (67%), and for marital status, (χ^2^ (3, *N* = 397) = 11.73, *p* = 0.008), with the majority of supportive care users (61%) being married or in a relationship. Differences were also found for place of residence (χ^2^ (1, *N* = 397) = 4.13, *p* = 0.04), with a greater proportion of people in the UK sample reporting supportive care use (34.4%) compared with the proportion in the Australian sample (22.3%).

In a comparison of the average time since diagnosis between groups, non-users of survivorship care services (*M* = 36.73, *SD* = 18.39) reported more time since diagnosis by 5.36 months (95% CI (1.10, 9.61)) than users (M = 31.38, SD = 18.68, *t*(395) = 2.48, *p* = 0.014, two-tailed, *d* = 0.29). Age also varied significantly between groups; non-users of survivorship support programmes (M = 64.58, SD = 11.98) were, on average, 7.67 years older (95% CI (4.70, 10.63)) than users of supportive care (*M* = 56.92, *SD* = 15.39, *t*(395) = 5.08, *p* < 0.001, two-tailed, *d* = 0.60).

Independent sample *t*-tests also tested whether the scores of personal control and emotional representations and use of problem-focused and emotion-focused coping differed between groups distinguished by supportive care use. The results are reported in Table 2. Users of survivorship support programmes indicated greater perceived personal control and also reported more use of both problem-focused and emotion-focused coping strategies, but no difference was observed between users and non-users of supportive care for emotional representations of cancer.

### 3.3. Influences on the Use of Survivorship Support Services

To test the study’s hypotheses and estimate the probability of supportive care use for cancer patients and survivors, two binary logistic regression analyses (with α = 0.05) were conducted. The likelihood of utilising survivorship support programmes was estimated from scores on personal control, emotional representations, and problem- and emotion-focused coping, after controlling for age, gender, time since diagnosis and marital status. Assumption testing conducted prior to the analyses did not indicate any violations.

The omnibus model for the first logistic regression analysis, which assessed whether personal control and problem-focused coping predicted the probability of supportive care use after controlling for demographics and time since diagnosis was statistically significant (χ^2^ (*df* = 7, *N* = 397) = 71.62, *p* < 0.001, Cox and Snell *R*^2^ = 0.17, Nagelkerke *R*^2^ = 0.25) (see Table 3). With the predictors entered, the model was 80% accurate in its predictions of supportive care use. The non-significant Hosmer and Lemeshow test results confirmed that the model involving personal control and problem-focused coping predicting uptake of survivorship support services was a good fit for the data (χ^2^ (*df* = 8, *N* = 397) = 14.87, *p* = 0.062). The model showed that only higher problem-focused coping was associated with a higher probability of supportive care use. Personal control was not significantly associated with a greater likelihood of using supportive care.

The omnibus model for the second logistic regression, which assessed whether emotional representations and emotion-focused coping predicted the probability of supportive care use after controlling for demographics and time since diagnosis, was also statistically significant (χ^2^ (*df* = 7, *N* = 397) = 58.78, *p* < 0.001, Cox and Snell *R*^2^ = 0.14, Nagelkerke *R*^2^ = 0.21) (see Table 4). With the predictors entered, the model was 78% accurate in its predictions of supportive care use. The non-significant Hosmer and Lemeshow test results confirmed that the model involving emotional representations and emotion-focused coping predicting uptake of survivorship support services was a good fit for the data (χ^2^ (*df* = 8, *N* = 397) = 5.19, *p* = 0.738).

As illustrated in Table 4, emotion-focused coping was the only predictor that significantly improved the model’s predictive capability. Higher adoption of emotion-focused coping was associated with greater use of survivorship support services. Emotional representations did not significantly influence the probability of a cancer patient or survivor using supportive care.

### 3.4. Does Emotion-Focused Coping Mediate the Relationship between Emotional Representations and Supportive Care Use?

On the bases of the non-significant relationship between emotional illness representations and supportive care use, and a linear regression that did not find a significant correlation between emotional representations and emotion-focused coping, a mediation model to test the hypothesis that the relationship between emotional representations and supportive care use may be mediated by emotion-focused coping was not conducted.

### 3.5. Does Problem-Focused Coping Mediate the Relationship between Personal Control and Supportive Care Use?

**Figure 1:**
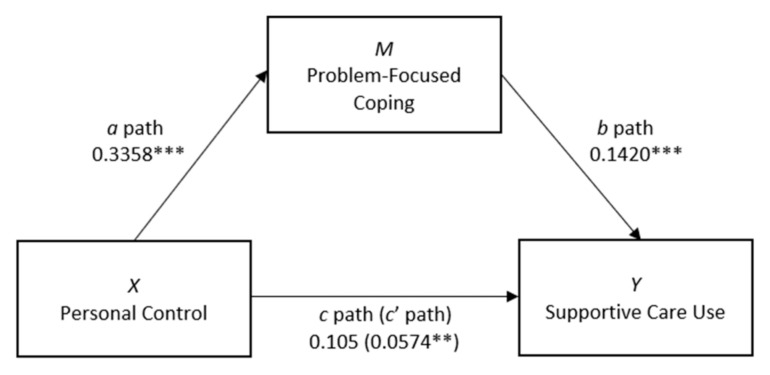
Path diagram of the mediation model predicting supportive care use from personal control and problem-focused coping. Note: Arrows indicate the hypothesised effects. Indirect (mediated) effect of *x* on *y* = *ab*. Direct (unmediated) effect of *x* on *y* = *c*’; *c* path = *ab* + *c*’. ** *p* < 0.05. *** *p* < 0.001.

The mediation model accounted for significant unique variance in the uptake of survivorship support programmes (*R*^2^ = 0.20, *F*(6, 386) = 16.08, *p* < 0.001). By Cohen’s [31] conventions, the combined effect of this magnitude can be considered ‘medium’ (*f*^2^ = 0.21). Step 1 of the model that regressed personal control on supportive care use, holding problem-focused coping consistent across patients (the *c*’ path), approached significance (*b* = 0.06, *se* = 0.03, *p* = 0.056), suggesting that cancer patients scoring higher for personal control were more likely to use survivorship support programmes (see Figure 1). Step 2 of the model showed that the regression of personal control on the mediator, problem-focused coping (the *a* path), was positive and statistically significant (*b* = 0.34, *se* = 0.04, *p* < 0.001). Step 3 found that the regression of problem-focused coping, controlling for personal control, on supportive care use (the *b* path) was positive and statistically significant (*b* = 0.14, *se* = 0.03, *p* < 0.001), indicating that cancer patients scoring higher on problem-focused coping are more likely to use survivorship support services than those scoring lower on the measure. Finally, the indirect effect of problem-focused coping on supportive care (*ab*) was tested using bootstrapping. Based on 5000 bootstrap samples, the indirect effect of problem-focused coping on supportive care use was found to be *b* = 0.05 (95% CI (0.02, 0.08), *se* = 0.01, *p* < 0.05), indicating the presence of significant mediation by problem-focused coping. Unstandardised (B) regression coefficients, 95% confidence intervals and *R*^2^ values for the mediation model are presented in Table 5.

## 4. Discussion

The aim of this study was to test the impact of personal control, emotional representations and problem- and emotion-focused coping on the use of survivorship support programmes by cancer patients and survivors. There was some support for the hypothesis that a subjective sense of personal control over cancer, more frequent use of problem-focused coping and greater adoption of emotion-focused coping predicted supportive care use; however, contrary to the hypothesis, cancer-related emotional representations did not. Further, as hypothesised, the results identified some support for the mediating role of problem-focused coping in the relationship between personal control and supportive care use. A mediation model predicting the uptake of survivorship support services from emotional representations via emotion-focused coping was not tested.

Consistent with much research using the Common Sense Model, the current study found that perceptions of the controllability of illness were positively associated with problem-focused coping [32,33]. In particular, there was some support for the hypothesis that users of supportive care would have a stronger sense of personal control over their cancer and would use problem-focused coping strategies more frequently than non-users. The utilisation of survivorship support programmes implies an active, open and strategic approach to cancer that extends beyond one’s immediate network [19]. Those who take advantage of support services are more likely to believe they have the power to influence their cancer and to cope by becoming behaviourally involved and seeking information about their illness [10]. As predicted by the Common Sense Model, the present results suggest that people with cancer who take the decisive step to engage with psychosocial support services not only perceive their cancer to be within their control but they are also more likely to adopt problem-focused coping procedures more readily, which, in turn, predicts supportive care use.

The hypothesis that more frequent use of emotion-focused coping in people with cancer would significantly predict supportive care engagement was also supported. Emotion-focused coping strategies describe attempts to regulate the inner turmoil caused by a stressor [34]. Survivorship support services are largely designed to provide informational and educational material as well as emotional support [2]. In availing themselves of psychosocial support services, patients and survivors can gain skills in learning how to live with and adapt to the reality of their illness while finding solace in the comfort and understanding of other patients and support care workers.

The lack of relationship observed between the emotional representations of cancer and supportive care use is consistent with some previous research. Skaczkowski et al. [2], for instance, found that oncology patients who reported higher levels of distress were less likely to take up referrals for survivorship support services. Similarly, Söllner et al. [35] observed that breast cancer patients’ distress (moderate or severe anxiety and/or depression) did not correlate with their utilisation of psychosocial support. The researchers speculated that patients may have had sufficient support from their existing familial and social networks. Alternatively, patients might have perceived expressing a need for external help as weakness. Of course, people who do not consider their distress sufficiently severe to warrant external help might not perceive any reason to seek out psychosocial care [36]. This corresponds with Fish and colleagues’ [37] study of the psychosocial barriers to help-seeking for cancer symptoms among Australian men, which found that when symptoms were felt to be less threatening, help-seeking was often delayed. When symptoms worsened with the passage of time, however, increasing levels of negative emotion precipitated a stronger desire to seek help. This, according to Fish et al., [37] is consistent with the Yerkes–Dodson law [38], in which moderate levels of stress are required for action. The association between emotional representations and supportive care use might comply more closely with an inverted-U shaped function. If so, low use of supportive care might indicate emotional reactions that are either slight, or so extreme that people try to avoid and deny the problem altogether.

Alternatively, a disinclination to seek out survivorship support programmes for emotional distress might signal a preference for privacy and self-help [2]. It is also possible that participants in the current study felt no cancer-specific distress but still availed themselves of supportive care services for other, non-cancer-related reasons. This is consistent with Söllner et al.’s [35] observation that a high percentage of oncology patients who do not identify as distressed still want to access psychosocial care.

The proposition in the Common Sense Model of the mediating role played by coping in the relationship between illness representations and behavioural outcomes was also examined. Reflecting past research [39,40], evidence in the present study that coping mediates the illness perception–behavioural outcome relationship was uneven. The results of a mediation model suggested that the relationship between personal control and supportive care use was partially mediated by problem-focused coping. However, statistical analyses conducted earlier in the study suggested no mediating role of emotion-focused coping in the relationship between emotional representations and supportive care use.

Although the findings that the utilisation of survivorship support services by individuals higher in personal control, problem-focused coping and emotion-focused coping were significant or approaching significance, these variables did not explain a high percentage of variance in supportive care use. This suggests that other contributing factors not considered in the present study were implicated in participants’ decision to take up supportive care or not in the preceding month. Marteau [41] emphasised the need to consider the effects on health behaviour of the broader environment within which an individual operates. Adding potential social and economic barriers, including family and work commitments, financial constraints and the availability of transport, to the battery of variables considered in this study might improve the current models’ explanatory power.

Demographic factors are likely to be important in the utilisation of supportive care. As with previous studies, our results confirmed that men were less likely to participate in programmes than women. This may reflect influence from perceptions of “masculinity” and associated stoicism or the belief that seeking support may threaten autonomy [42]. Attempts to address the gender imbalance could focus on challenging these beliefs or exploring strategies for improving the “acceptability” of supportive services by targeting the content and mode of delivery designed to meet the needs of male survivors. Further, place of residence (Australia or the UK) was a significant influence on supportive care use, with a greater proportion of those who resided in the UK reporting greater use of supportive care. This may, in part, be due to different supportive care services available in the UK compared with Australia. Although dedicated centres that provide information and support to people with cancer have emerged in some Australian cancer centres [43], across the UK, there are currently over 22 Maggie’s Centres located in the grounds of specialist NHS cancer hospitals that provide informational, practical, social and emotional support to people with cancer (www.maggies.org; accessed on 5 April 2021). Increased availability and accessibility of survivorship support programmes and services have the potential to promote greater use in people with cancer. It is likely that a range of attitudinal variables impact support service utilisation by people with cancer regardless of gender. McDowell et al. [44], for example, found that cancer patients from a regional cancer treatment centre in Queensland, Australia, with more positive attitudes towards help-seeking were more likely to utilise support services. Attitudes to specific types of support also appear to influence engagement with psychosocial support services. In their analysis of the factors associated with the offer and use of supportive care referrals in a large metropolitan hospital with in-house supportive care services in Melbourne, Australia, Skaczkowski and colleagues [2] discerned a relationship between the kind of referral offered and that taken up by oncology patients. Support services providing physical treatment (e.g., massage) were the most likely to be accepted by patients (87%), while referrals to services providing emotional support were the least likely to be accepted (53%).

Although no specific predictions were made regarding which patient characteristics might be associated with support service use, the study found that certain clinical factors were statistically significant. On average, users of survivorship support programmes were found to have received their cancer diagnosis more recently than non-users. This is likely to reflect a decrease in informational and emotional needs among patients who have completed treatment and moved further into the recovery phase. However, as McDowell et al. [45] contended, many people still have psychosocial care needs as they enter and move through the long-term survivorship phase of the cancer journey.

Further, use of survivorship support services may be impacted by the availability of services that address relevant survivorship needs of individuals. For example, in a recent review of reviews, Crawford-Williams and colleagues [46] found that despite the American Cancer Society publishing prostate cancer survivorship guidelines, existing survivorship interventions did not address key survivorship domains (e.g., cancer surveillance and care coordination). The review also highlighted that survivorship interventions were poorly evaluated in the literature and that methods for scaling the interventions to large populations were poorly described. Thus, there is the potential that some individuals seek survivorship interventions that are currently not offered across health services or the community.

### 4.1. Limitations

Although cross-sectional studies are useful “snapshots” of the relationships between variables at a particular moment, they do not provide conclusive evidence of a causal direction in relationships. In seeking to meet the physical, emotional and psychological needs of individuals living with or affected by cancer, supportive care is designed to help build patients’ sense of personal control over their illness [47]. Consequently, it is possible that supportive care use heightened a sense of personal control and problem-solving among participants, as opposed to these factors driving participation.

Even when survivorship support services are offered to oncology patients, studies have found poor engagement with them, with reports in Australia indicating 25% [48] and 38% [49] of patients declining referrals [2]. As many as 75% of participants in the present study reported not engaging with any psychosocial support programmes. This could be the result of a methodological limitation regarding the time frame in which participants were assessed. Those eligible to take part in the current study had to have been diagnosed with cancer in the 5 years preceding survey completion. However, participants were only asked to indicate whether they had made use of any psychosocial care services in the previous month. Consequently, it is possible that participants in the current study may have developed intentions to use supportive care that were not captured at the time of survey completion. Additionally, participants might have accessed survivorship support services more than a month prior to undertaking the survey.

Another limitation of the present study is that participants were not asked to indicate whether they were aware of the availability of psychosocial support services and, for those who were not, whether they would have utilised them if they had known of their existence. Insufficient knowledge of available supportive care has been identified as a barrier to service utilisation in other studies [50], making it an important area of investigation in predicting supportive care use. Further, no inquiries were made in the present study regarding participants’ beliefs about supportive care, nor were patients invited to comment on why they used or did not use survivorship support services.

Finally, it is possible that other aspects of illness perceptions are associated with seeking supportive care (e.g., illness coherence), and that these might be related to coping strategies. However, the targeted variables were viewed as the key drivers of seeking supportive care.

### 4.2. Future Research

To capture the dynamic nature of the Common Sense Model, prospective studies should adopt a longitudinal approach to facilitate a more detailed and nuanced exploration of pathways to supportive care use [26]. Reactions to illnesses are mutable, changing with illness progression and fluctuations in context. Specifically, an individual’s sense of personal control and emotional representations of cancer are likely to alter with the passage of time, as will their coping strategies and health care behaviours. In addition, examining whether other aspects of illness perceptions, such as illness coherence and impacts on survivorship care use, may increase our understanding of the drivers of supportive care utilisation.

Future research should consider whether patients make use of survivorship support services at any time during their cancer experience, and whether specific services are more or less desirable at different time points following diagnosis and treatment. This is particularly important, given the findings that unmet psychosocial care needs persist over time for many patients as they enter and go through the survivorship phase of the cancer trajectory [44,45,51]. In addition, the development, evaluation and implementation of survivorship services that comprehensively capture the key domains identified in existing survivorship guidelines [46] have the potential to meet the needs of people with cancer across the cancer trajectory.

## 5. Conclusions

An individual’s subjective sense of personal control over cancer is associated with supportive care use, but their cancer-specific emotional representations are not. This suggests that the Common Sense Model may provide a more useful framework for understanding the role that cognitive, rather than affective, illness appraisals play in predicting an individual’s decision to uptake supportive care services or not. Both higher problem-focused and emotion-focused coping predicted engagement with survivorship support services. Utilising both kinds of coping to a higher degree might help oncology patients deal with the diagnosis and treatment of cancer more effectively than invoking either coping strategy on its own.

Given the absence in the current sample of a linear relationship between emotional representations and emotion-focused coping, a model testing whether emotion-focused coping mediated the relationship between emotional representations and supportive care use was not conducted. Problem-focused coping was found to partly mediate the relationship between personal control and supportive care use. However, greater clarity about the role of coping in relation to illness perceptions is required if we are to apply this information to the design of interventions formulated to increase supportive care uptake among people with cancer. Findings from the current study have the potential to stimulate future research and theory development that advance our understanding of the processes by which cognitive and affective beliefs impact coping and health outcomes in chronic illness. Additional research efforts should continue to consider why most patients do not utilise psychosocial care services despite the considerable health benefits derived from their uptake and the significant distress associated with the diagnosis and treatment of cancer.

## Figures and Tables

**Table 1:** Demographics, cancer diagnosis, years since diagnosis, and supportive care use of participants.

Sociodemographic Characteristic	*n*	%	Age	
			Mean	Standard Deviation
Gender				
Male	191	51.9	67.65	10.35
Female	206	48.1	58.29	14.02
Education				
Primary school	3	0.8	-	-
High school (years 7–10)	55	13.9	-	-
High school (years 11–12)	61	15.4	-	-
University (undergraduate)	92	23.2	-	-
University (postgraduate)	47	11.7	-	-
Vocational	139	35.0	-	-
Marital status				
Single	52	13.1	-	-
In a relationship	262	66.0	-	-
Divorced	55	13.9	-	-
Widowed	28	7.1	-	-
Cancer type				
Breast	91	22.9%	-	-
Colorectal	31	7.8%	-	-
Lung	15	3.8%	-	-
Haem	24	6.0%	-	-
Prostate	75	18.9%	-	-
Melanoma	33	8.3%	-	-
CNS	1	0.3%	-	-
Other	127	32.0%	-	-
Years since diagnosis				
Up to 1 year	49	12.3	-	-
1–2 years	79	19.9	-	-
2–3 years	80	20.2	-	-
3–4 years	62	15.6	-	-
4–5 years	93	23.4	-	-
5+ years	34	8.6	-	-
Supportive care use				
Did not use	300	75.8	-	-
Did use	97	24.2	-	-

**Table 2:** Independent sample *t*-tests comparing mean personal control, emotional representations, problem-focused coping and emotion-focused coping scores by supportive care use.

Independent Variables	Score Range	Supportive Care Users		Supportive Care Non-users		*t*(395)	*p*	Cohen’s *d*
		M	SD	M	SD			
Personal Control	6–30	20.64	4.70	18.76	5.32	−3.08	0.002	0.36
Emotional Representations	6–30	18.43	5.89	17.24	6.37	−1.60	0.111	0.19
Problem-Focused Coping	6–24	16.02	3.86	12.63	4.29	−6.90	<0.001	0.81
Emotion-Focused Coping	10–40	24.43	5.38	20.73	5.28	−5.94	<0.001	0.70

**Table 3:** Personal control and problem-focused coping as predictors of supportive care use.

Predictor Variables	*b*	*SE*	*p*	*Exp*(*B*) (95% CI)
Constant	−1.16	-	-	-
Personal Control	0.06	0.03	0.056	1.06 (1.00, 1.12)
Problem-Focused Coping	0.14	0.03	<0.001	1.15(1.07, 1.23)

*b* = unstandardised regression coefficient; *SE* = Standard Error; *p* = *p*-value; *Exp(B)* = odds ratio; 95% CI = 95% confidence interval.

**Table 4:** Emotional representations and emotion-focused coping as predictors of supportive care use.

Predictor Variables	*b*	*SE*	*p*	*Exp*(*B*) (95% CI)
Constant	−0.74	-	-	-
Emotional Representations	0.00	0.03	0.921	1.00 (0.96, 1.05)
Emotion-Focused Coping	0.11	0.03	<0.001	1.12 (1.06, 1.17)

**Table 5:** Mediation model for personal control, problem-focused coping and supportive care use.

Variable	*B* (95% CI)	*SE* (HC3)
DV = Problem-Focused Coping (*R*^2^ = 0.20 ***)		
Constant	11.14 (8.15, 14.13)	1.42
Personal Control	0.34 (0.26, 0.41) ***	0.04
DV = Supportive Care Use (Cox–Snell = 0.17, Nagelkerke = 0.25 ***)		
Constant	−1.16 (−3.08, 0.76)	0.98
Personal Control	0.06 (−0.006, 0.17)	0.03
Problem-Focused Coping	0.14 (0.07, 0.21) ***	0.03

CI = confidence interval; DV = dependent variable; *SE* = Standard Error *** *p* < 0.001.

## Data Availability

The data presented in this study are available on request from the corresponding author. The data are not publicly available due to privacy and ethical considerations.
